# Prediction model of adnexal masses with complex ultrasound morphology

**DOI:** 10.3389/fmed.2023.1284495

**Published:** 2023-12-07

**Authors:** Yuqing Wu, Kuo Miao, Tianqi Wang, Changyu Xu, Jinlai Yao, Xiaoqiu Dong

**Affiliations:** Department of Ultrasound, The Fourth Affiliated Hospital of Harbin Medical University, Harbin, China

**Keywords:** ultrasound, adnexal masses, O-RADS, nomogram, prediction model

## Abstract

**Background:**

Based on the ovarian-adnexal reporting and data system (O-RADS), we constructed a nomogram model to predict the malignancy potential of adnexal masses with sophisticated ultrasound morphology.

**Methods:**

In a multicenter retrospective study, a total of 430 subjects with masses were collected in the adnexal region through an electronic medical record system at the Fourth Hospital of Harbin Medical University during the period of January 2019–April 2023. A total of 157 subjects were included in the exception validation cohort from Harbin Medical University Tumor Hospital. The pathological tumor findings were invoked as the gold standard to classify the subjects into benign and malignant groups. All patients were randomly allocated to the validation set and training set in a ratio of 7:3. A stepwise regression analysis was utilized for filtering variables. Logistic regression was conducted to construct a nomogram prediction model, which was further validated in the training set. The forest plot, C-index, calibration curve, and clinical decision curve were utilized to verify the model and assess its accuracy and validity, which were further compared with existing adnexal lesion models (O-RADS US) and assessments of different types of neoplasia in the adnexa (ADNEX).

**Results:**

Four predictors as independent risk factors for malignancy were followed in the preparation of the diagnostic model: O-RADS classification, HE4 level, acoustic shadow, and protrusion blood flow score (all *p* < 0.05). The model showed moderate predictive power in the training set with a C-index of 0.959 (95%CI: 0.940–0.977), 0.929 (95%CI: 0.884–0.974) in the validation set, and 0.892 (95%CI: 0.843–0.940) in the external validation set. It showed that the predicted consequences of the nomogram agreed well with the actual results of the calibration curve, and the novel nomogram was clinically beneficial in decision curve analysis.

**Conclusion:**

The risk of the nomogram of adnexal masses with complex ultrasound morphology contained four characteristics that showed a suitable predictive ability and provided better risk stratification. Its diagnostic performance significantly exceeded that of the ADNEX model and O-RADS US, and its screening performance was essentially equivalent to that of the ADNEX model and O-RADS US classification.

## 1 Introduction

Ovarian tumors are common tumors of the female reproductive system (1), and most patients with ovarian malignancies are already in the middle to late stages at the time of diagnosis and lack ideal treatment outcomes. Ovarian tumors are ranked as the second most common cause of cancer-related death in gynecological disease, with a 5-year survival rate of less than 40% (2). According to studies, borderline ovarian tumors (BOTs) are potential malignancy ovarian tumors, and peritoneal metastases are present in 10% of bots, which are often overlooked and misdiagnosed in studies. Frozen pathological samples tend to diagnose BOTs as benign tumors in 25–30% of cases and identify BOTs as carcinoma in 20–30% of cases (3). The boundary between borderline ovarian tumors and benign and malignant tumors is not clear-cut. Therefore, it is critical to determine the nature of the mass in women with lesions in the adnexal area (4). Since the mid-80s, ultrasound has become the main imaging procedure for evaluating lesions in the adnexal region (5); it not only facilitated detailed observations of the location, size, morphology, composition, and blood flow of ovarian tumors but also facilitated simple, low-cost, and radiation-free test procedures as well as reproducible results. However, the complex presentation of ovarian tumors and the prevalence of same-picture heterogeneous manifestations resulted in large differences between different physicians’ diagnoses (6). In this study, cystic or solid adnexal zone lesions with complex ultrasound morphologic features such as ascites, irregular internal wall, irregular morphology, abundant blood flow, and multiple papillae (fulfilling one will suffice) were termed complex lesions in the adnexal zone.

Recently, scholars have proposed various guidelines for the evaluation of ovarian-adnexal masses, including simple rules (SRs) (7), risk of malignancy index (RMI) (7, 8), and gynecology imaging reporting and data system (GI-RADS) (9, 10). RMI is the most frequently validated model. The logistic regression model LR2, developed by the International Ovarian Tumor Analysis (IOTA) study, with a risk cutoff of 10% and SR, is by far the most commonly used model for predicting the benign and malignant nature of ovarian masses. However, these guidelines were inefficient at solving the abovementioned problems and had very limited international acceptance. In 2020, the American College of Radiology (ACR), after joint discussions between multidisciplinary experts (including gynecologists and ultrasound specialists), consolidated ultrasound standards (based upon those already introduced) and officially published the ovarian-adnexal imaging reporting and data system (O-RADS) for ultrasound risk stratification and management, with reference to the International Ovarian Tumor Analysis (IOTA) ADNEX Model consensus guidelines (11); this unified the standardized ultrasound terminology, reduced ambiguity in ultrasound reporting, and provided treatment guidelines for ovarian lesions. However, O-RADS led to high diagnostic sensitivity, low specificity (9), and overtreatment in clinical use problems, which may cause a cumbersome and time-consuming assessment process. Moreover, the probability of O-RADS 4 malignancy is 10–50%, which is a wide range and not conducive to the precise clinical management of Category 4 tumors. Currently, many scholars have conducted studies on the column charts associated with ovarian complex lesions in order to optimize the O-RADS model, improve the accuracy and clinical utility of the O-RADS, and simplify the O-RADS evaluation process, which has demonstrated the accuracy of the clinical prediction model (12). Among them, Gong developed a column chart for identifying complex lesions in the adnexal region (13), but since the model was a single-center study and the included population only included patients who underwent gynecological surgery, human bias was difficult to avoid, and in addition, on the basis of Gong, the present study added novel indicators, such as tumor marker HE4 (14), which resulted in a more comprehensive extraction of features and a more accurate acquisition of independent risk factors.

In this study, we constructed a comprehensive prediction model based on O-RADS, combining clinical features, laboratory tests, and other ultrasound indicators, with the aim of improving the non-invasive assessment of the benign and malignant nature of complex lesions in the adnexal region, assisting in the clinical diagnosis of the disease with greater precision, and developing personalized treatment management strategies in order to improve the diagnostic performance of junior ultra-sonographers.

## 2 Methods

### 2.1 Study subjects

**Figure 1:**
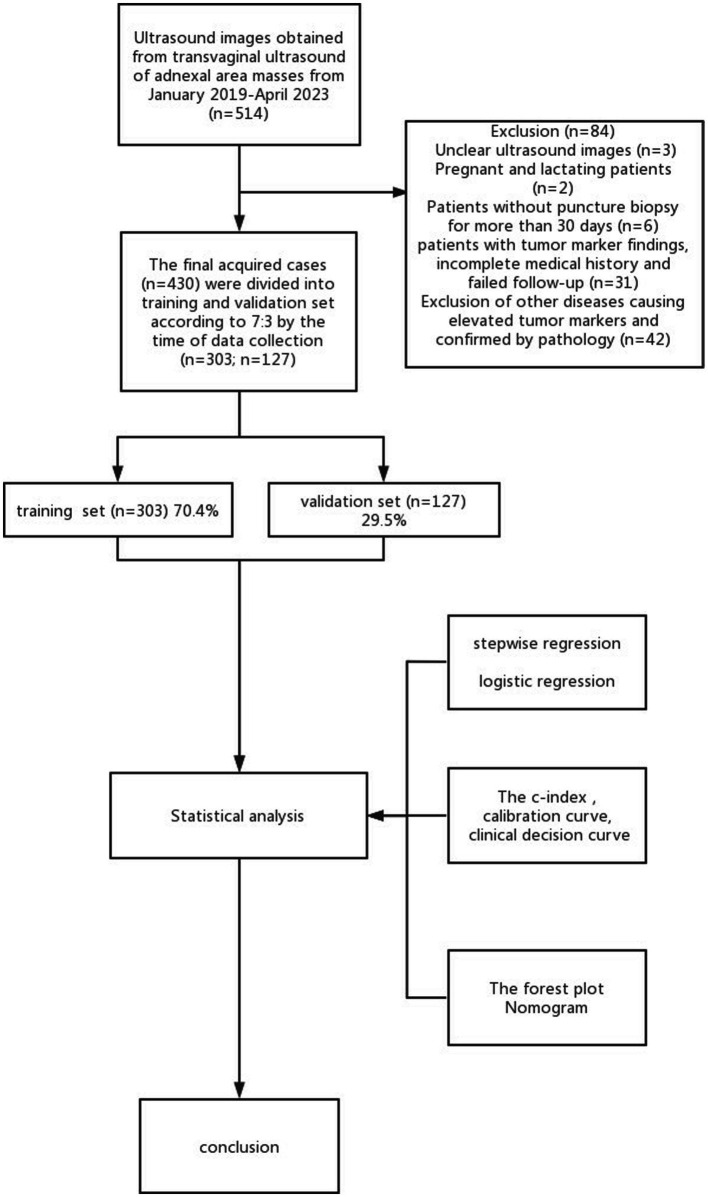
Flowchart of research subjects.

Retrospectively analyzing 430 cases of pathologically confirmed ovarian tumors (confirmed from January 2019 to April 2023), 303 cases were included in the training set and 127 in the validation one, obtaining a 7:3 ratio. Based on the postoperative pathological findings, these cases were subdivided into benign and malignant groups. Because the management of functional ovarian tumors requires reference to malignant tumors, the junction’s tumors were classified as malignant. The inclusion criteria were as follows: (1) the lesion was histopathologically evaluated after surgical resection, and the time interval between ultrasonography and surgery did not exceed 30 days; (2) presence of at least one adnexal complex lesion in the solid or cystic adnexal area probed by transnational and/or transfiguring ultrasonography; (3) all patients underwent translational and/or transfiguring ultrasonography, CDFI, and tumor markers before surgery. The exclusion criteria were as follows: (1) patients during pregnancy and lactation; (2) patients who did not undergo tumor series; (3) poor quality of ultrasound images or incomplete ultrasound evaluation; (4) other diseases causing abnormal elevation of serum CA125 and HE4 (Figure 1) (13). This study was subject to approval by the medical ethics committee, and all patients provided informed consent.

### 2.2 Research design and methodology

#### 2.2.1 Data collection

#### 2.2.2 Clinical data

Patients’ ages, preoperative CA125 levels, HE4 levels, CA199 levels, family histories of ovarian cancer, risk of ovarian malignancy algorithm (ROMA), and human papillomavirus (HPV) infection histories were collected as quantifiable baseline data. Women aged 20–30 years, 30–60 years, and > 60 years were classified into adolescence, fertility, and menopause groups.

##### 2.2.2.1 Ultrasonography

A GE Logiq E9 color Doppler ultrasound diagnostic instrument with a RIC5-9-D intracellular probe and a C1-5-D transfiguring convex array probe with frequencies of 5.0–9.0 MHz and 1.0–5.0 MHz, respectively, was used to scan the uterus, bilateral adnexal region, and pelvis in transverse, longitudinal, and oblique views. Two-dimensional ultrasound features such as solidity, boundary, morphology, internal egocentricity, the presence or absence of separation, acoustic shadow, protrusion structures, ascites, and peritoneal nodules were recorded; and the color blood flow sampling frame should contain solid lesions, and the section with the highest number of vessels was selected to observe the blood flow signal and the location after image stabilization. According to the criteria of the IOTA group (15), the color scoring of the blood flow signal in the solid protrusions proceeded with a score of 1–4 representing no blood flow, minimal blood flow, moderate blood flow, and significant blood flow, respectively. If the mass was large and its contour was difficult to show using translating ultrasound, a combination of transfiguring ultrasound and translational ultrasound was required. The collected images were obtained by two ultra-sonographers with more than 5 years of experience along with the chief of the ultrasound department.

##### 2.2.2.2 Laboratory-related tests

To prepare for the examination, 5 mL of venous blood was drawn on an empty stomach. According to the Guide Standards for Tumor Markers, the normal values of CA125, HE4, and CA199 were 0–35u/mL, 0–105.69u/mL, and 0–30u/mL (16), and the specific values were classified as routine, 1-fold elevated, 2-fold elevated, and more than 3-fold elevated, respectively.

#### 2.2.3 Model reference standards

##### 2.2.3.1 O-RADS score and model evaluation criteria

The cystic solidity, boundary, morphology, blood flow signal, internal echogenicity, protrusion, and contents of the mass were to symbolize each pelvic lesion that was placed in one of the categories (O-RADS 0–5) and stratify the risk according to the flowchart: (1) O-RADS category 0: lesions that cannot be fully evaluated using ultrasonography; (2) O-RADS category 1: physiological category, referring to normal premenopausal ovaries; (3) O-RADS category 2: almost certainly benign lesions (malignant risk <1%); (4) O-RADS 3 category: low-risk (malignant risk of ≥1, <10%); (5) O-RADS 4 category: intermediate risk (malignant risk of ≥10, <50%); (6) O-RADS 5 category: high risk (malignant risk of ≥50%) (17). In this study, the risk threshold for malignancy was identified as 10% (18) when comparing benign and malignant; that is, an O-RADS Category 4 or higher was defined as malignant.

##### 2.2.3.2 ADNEX risk and model evaluation criteria

The ADNEX model was acquired on the IOTA website.^1^ The model consisted of nine predictors, comprising six ultrasound variables and three clinical information: subject age, serum CA 125 level, type of center, maximum diameter of the lesion, maximum diameter of the largest solid component of the lesion, more than 10 cyst locules, number of protrusion projections, presence of acoustic shadows, and presence of ascites (19). In the ADNEX model, the risk of malignancy was expressed by a percentage and histogram. Since the optimal cutoff value of the model was flexible, the present study defined the malignancy risk threshold as 10% in the ADNEX model assessment (less than 10% as benign and more than 10% as malignant) (18).

##### 2.2.3.3 Reference standard

Postoperative histopathology as a criterion for diagnosing benign and malignant masses was published in the pathology department. The diagnostic criteria were based on the World Health Organization (WHO) guidelines, and the tumor classification was done based on the guidelines of the International Federation of Gynecology and Obstetrics (FIGO) (20, 21).

## 3 Statistical analysis

All statistical analyses were performed by utilizing the IBM-SPSS (version 27.0) and the R software. The 430 adnexal masses with complex ultrasound morphology were grouped in a training set of 303 masses and a validation set of 127 masses based on the date of data collection for validation, consistent with a theoretical 7:3 ratio (22). Continuous and categorical variables between training and validation sets were analyzed using a *t*-test and chi-square test, respectively. Variables were unpacked utilizing university logistic regression. The stepwise regression method was used to filter variables and select statistically significant indicators to construct a nomogram (23, 24). We evaluated the model from three aspects, which included accuracy, discriminatory ability, and clinical utility. The predictive power of the three models (ADNEX model, O-RADS US, and our nomogram model) was evaluated by calculating the C-index. To reduce the bias, calibration curves and the Hosmer–Lemeshow test were used to evaluate the consistency of the model (25). AUC under the ROC curve was utilized to assess the discriminatory power of the model. The decision curve analysis was used to assess the clinical validity of the model by reckoning the net benefit (25).

## 4 Results

### 4.1 Participant characteristics

A total of 430 patients with 430 adnexal lesions were enrolled in this study between January 2019 and May 2023, of which there were 245 benign and 185 malignant adnexal lesions (see Table 1). Benign adnexal lesions accounted for 57% of cases, with the most common benign lesion being cystic teratoma (16%). Malignant lesions in the adnexal region accounted for 43% of cases, with the most common histologic type being high-grade plasma cystadenocarcinoma (33%) (Appendix 1). The mean ages of the adnexal benign and malignant groups were 42.38 ± 15.05 and 52.07 ± 13.35 years, respectively; the CA125 levels were 38.6 ± 5.6 and 240 ± 60.4 U/mL, respectively; and the HE4 levels were 63 ± 10.3 U/mL and 176.3 ± 20.9 U/mL, respectively. The difference was statistically significant. Among the malignant adnexal lesions, 64% were found in menopausal women, which was higher than that in the benign group; 43.5% were in fertile women, which was slightly lower than that in the benign group; and 19% were in pubertal women, which was significantly lower than that in the benign group. The differences were statistically significant. Standard tumor marker levels accounted for 20–30% of the cases, and those with levels elevated two times or more accounted for 50–60% of cases, which was statistically significant when compared with that of the benign group. The O-RADS categories 2, 3, 4, and 5 accounted for 0, 0.05, 48, and 94.7% of cases, respectively, and the difference was statistically significant compared with the benign group. The blood flow scores of acoustic shadows and papillae were higher than those of the benign lesion group, and the difference was statistically significant (*p* < 0.05, see Table 2).

**Table 1:** Pathological findings of 430 adnexal tumors.

Pathology	Internal validation (*n* = 430)	External validation (*n* = 157)
Benign	*n* = 245	*n* = 94
Endometrioma	33 (13%)	13 (14%)
Ovarian abscess	26 (11%)	10 (9%)
Luteal cyst	27 (11%)	5 (5%)
Hematoma luteal	17 (7%)	7 (7%)
Benign cystic teratoma	38 (16%)	16 (17%)
Serous cystadenoma	29 (12%)	11 (12%)
Mucinous cystadenoma	31 (13%)	14 (15%)
Plasma mucous cystadenoma	6 (2%)	2 (2%)
Follicular Membranous Cell Tumor	21 (9%)	7 (7%)
Fibroma	6 (2%)	3 (3%)
Sertoli-Leydig tumor	4 (2%)	3 (3%)
Brenner tumor	7 (3%)	3 (3%)
Malignant	*n* = 185	*n* = 63
Junctional Plasmacytoid papilloma	7 (4%)	0
Junctional Serous cystadenoma	8 (4%)	2 (3%)
Junctional Mucinous cystadenoma	10 (5%)	1 (2%)
Junctional Mixed tumor	3 (2%)	0
Junctional Endometrioid carcinoma	4 (2%)	2 (3%)
Clear cell cancer	29 (16%)	14 (22%)
High-grade serous ovarian carcinoma	33 (18%)	10 (16%)
Plasmacytoid protrusion carcinoma	19 (10%)	7 (11%)
Mucinous neoplasm	26 (14%)	12 (19%)
Endometrioid carcinoma	17 (9%)	5 (8%)
immature teratoma	5 (3%)	3 (5%)
Granulosa cell tumor	11 (6%)	2 (3%)
Metastatic tumor	9 (5%)	3 (5%)
Mixed tumor	4 (2%)	2 (3%)

Simple unilocular cysts have been excluded in this study. Junctional tumors are potentially malignant and were classified as malignant in this study.

**Table 2:** Clinical and ultrasound characteristics of patients with adnexal masses in the training and validation sets.

Indicators	Pathology (Training cohort) (*n* = 303)			Pathology (Validation cohort) (*n* = 127)			*p*
	Benign	Malignant	Total	Benign	Malignant	Total	
Clinical features							
Age (years)							0.001^b^
Adolescence	37	7	44	16	3	19	
Fertility	114	84	198	51	38	89	
Menopause	21	40	61	5	14	19	
Ca125 U/mL (Average range)							0.001^b^
Normal	127	74	201	58	30	88	
Up 1X	29	17	46	11	8	19	
Up 2X	6	7	13	1	5	6	
Up 3X	10	33	43	2	12	14	
HE4 U/mL (Average range)							0.000^b^
Normal	163	77	240	65	45	100	
Up 1X	8	17	25	5	5	10	
Up 2X	1	14	15	0	6	6	
Up 3X	11	12	23	2	7	11	
Ca199 U/mL (Average range)							0.000^b^
Normal	143	95	238	58	29	87	
Up 1X	17	18	35	11	8	19	
Up 2X	6	8	14	1	5	6	
Up 3X	6	10	16	2	13	15	
OV Genetic History							0.078^b^
Yes	26	29	55	11	12	23	
No	146	102	248	60	44	104	
HPV Infection							0.677^b^
Yes	18	16	34	8	6	14	
No	154	115	269	65	48	113	
ROMA							0.000^b^
Low risk	113	45	158	49	20	69	
High risk	59	86	145	25	33	58	
Ultrasound characteristics							
O-RADS							0.000^b^
2	40	0	40	11	0	11	
3	49	2	51	21	2	23	
4	80	84	164	39	26	65	
5	3	4	48	27	1	28	
> 3 papillations							0.000^b^
Yes	28	77	105	12	31	43	
No	144	54	198	61	23	84	
Color score							0.000^b^
1–2 score	164	90	254	70	36	106	
3–4 score	8	41	49	3	18	21	
Present as ascites/(peritoneal nodules)							0.000^b^
Yes	29	43	72	14	16	30	
No	143	88	231	62	35	97	
Irregular inner wall							0.007^b^
Yes	87	107	194	38	45	83	
No	85	24	109	35	9	44	
Acoustic shadowing							0.001^b^
Yes	111	25	136	29	11	40	
No	61	106	167	44	43	87	
Protrusion blood flow							0.000^b^
Yes	37	89	126	16	36	52	
No	135	42	177	57	18	75	
Maximum dimension of lesion (cm) (IQR)	7.5 (5.3, 9.8)	9.2 (6.1, 14.0)		7.5 (5.3, 9.5)	9.0 (5.3, 9.5)		0.000^c^
Maximum diameter of solid tissue (cm) (IQR)	0 (0, 2.7)	4.6 (2.8, 6.6)		0 (0, 2.7)	4.5 (2.5, 6.4)		0.000^c^

^b^is the χ^2^ test ^c^ is the rank sum test. χ^2^ for normal distribution; z for non-normal distribution. IQR, interquartile range; O-RADS, Ovarian Adnexal Reporting and Data System; CA 125, Cancer Antigen 125; CA199, cancer antigen 199; HE4, Human epididymis gene product 4.

### 4.2 Feature selection

Univariate analysis of the training set showed that the differences in age, CA125, CA199, ROMA, and HE4 levels, O-RADS score, acoustic shadow, ascites and peritoneal nodules, and blood flow scores of papillary protuberances were statistically significant when comparing the malignant and benign groups (all *p* < 0.05), but the differences in family history of ovarian tumors, and history of HPV infection were not (all *p* > 0.05, see Table 3).

**Table 3:** Univariate and multivariate analyses of ultrasound characteristics.

Indicators	Pathology		Univariate analysis		Multivariate analysis	
	Benign	Malignant	OR	*p*	OR	*p*
Clinical features						
Age			2.75 (1.80, 4.21)	<0.001	0.96 (0.48, 1.92)	0.92
Adolescence	43 (25%)	10 (7.6%)				
Fertility	109 (63.4%)	84 (64.1%)				
Menopause	20 (11.6%)	37 (28.2%)				
ca125U/mL (Average range)			1.67 (1.33, 2.10)	<0.001	0.96 (0.59, 1.55)	0.88
Normal	127 (73.8%)	74 (56.5%)				
Up 1X	29 (16.9%)	17 (13%)				
Up 2X	6 (3.5%)	7 (5.3%)				
Up >3X	0 (5.8%)	33 (25.2%)				
HE4 U/mL (Average range)			4.26 (2.55, 7.13)	<0.001	3.99 (1.99, 8.01)	<0.001
Normal	163 (67.9%)	77 (32.1%)				
Up 1X	7 (28%)	18 (72%)				
Up 2X	0 (0%)	15 (11.5%)				
Up>3X	2 (8.7%)	21 (91.3%)				
Ca199U/m (Average range)			1.39 (1.04, 1.87)	0.02	0.94 (0.57, 1.55)	0.81
Normal	143 (83.1%)	95 (72.5%)				
Up 1X	17 (9.9%)	18 (13.7%)				
Up 2X	6 (3.5%)	8 (6.1%)				
Up >3X	6 (3.5%)	10 (7.6%)				
OV Genetic History			1.60 (0.89, 2.87)	0.12		
Yes	26 (15.1%)	29 (22.1%)				
No	146 (84.9%)	102 (77.9%)				
HPV Infection			1.19 (0.58, 2.43)	0.63		
Yes	18 (10.5%)	16 (12.2%)				
No	154 (89.5%)	115 (87.8%)				
Ultrasound Characteristics			13.09 (7.55, 22.72)	<0.001	12.74 (6.56, 24.78)	<0.001
O-RADS						
2	40 (73.3%)	0 (26.6%)				
3	49 (96%)	2 (4%)				
4	80 (48.7%)	84 (51.3%)				
5	3 (6.3%)	45 (95.7%)				
Acoustic shadowing			0.16 (0.09, 0.3)	<0.001	0.23 (0.086, 0.63)	0.004
Yes	79 (83.2%)	16 (16.8%)				
No	93 (44.7%)	115 (55.3%)				
Protrusion blood flow			7.46 (4.46, 12.49)	<0.001	2.33 (1.02, 5.32)	0.04
Yes	37 (29.6%)	88 (70.4%)				
No	135 (75.8%)	43 (24.2%)				
Present as ascites /(peritoneal nodules)			2.4 (1.4, 4.1)	0.01	2.0 (0.95,5.02)	0.067
Yes	29	43				
No	143	88				
ROMA			3.66 (2.27, 5.9)	<0.001	2.18 (0.96,4.12)	0.062
Low risk	113	45				
High risk	59	86				

### 4.3 Model construction

**Figure 2:**
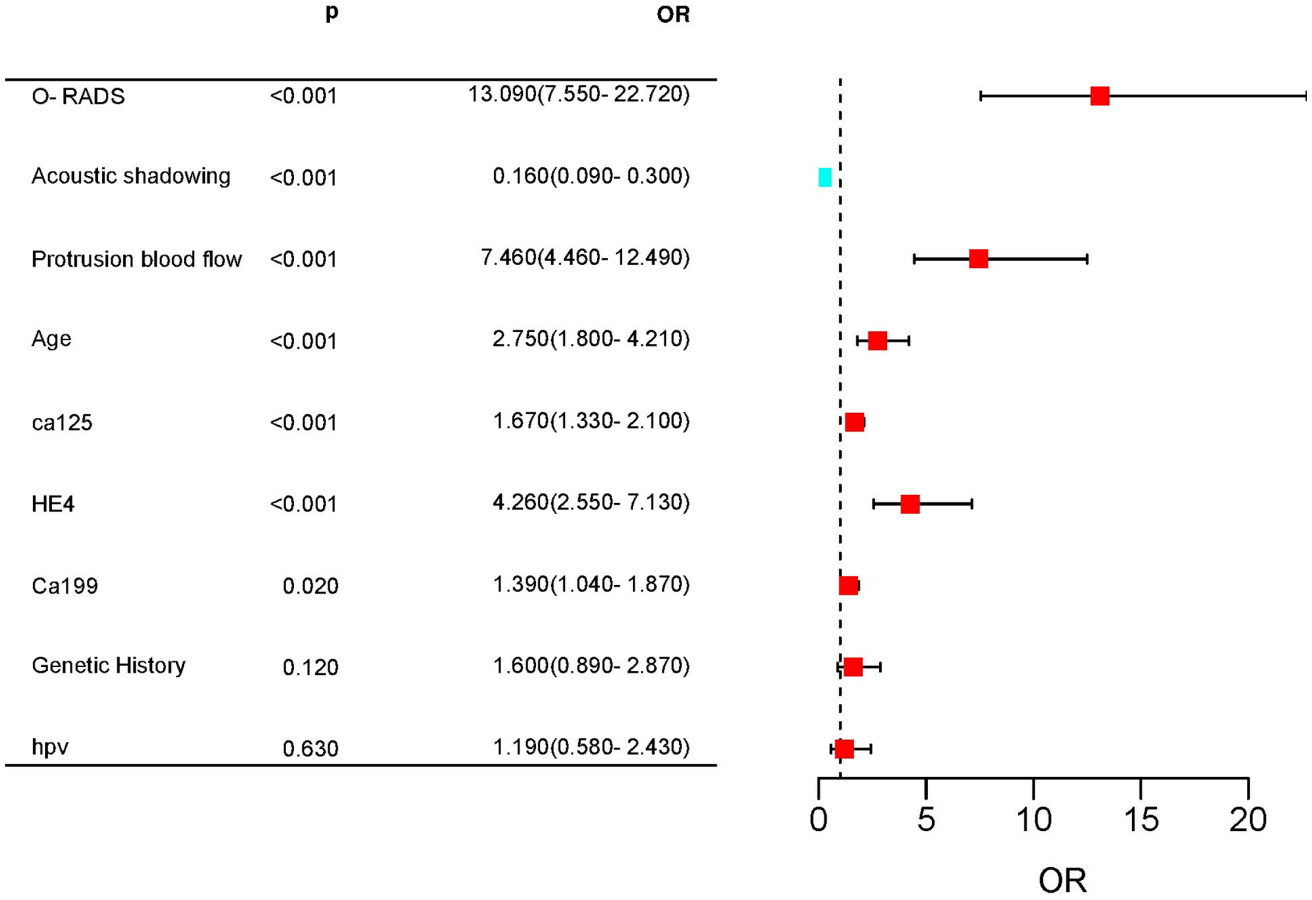
Forest plot for predicting complex lesions in the adnexal region based on univariate Cox regression. Age, CA125, HE4, CA199, ORADS score, acoustic shadow, and blood flow score of protrusions were highly significant differences between the malignant and benign groups.

**Figure 3:**
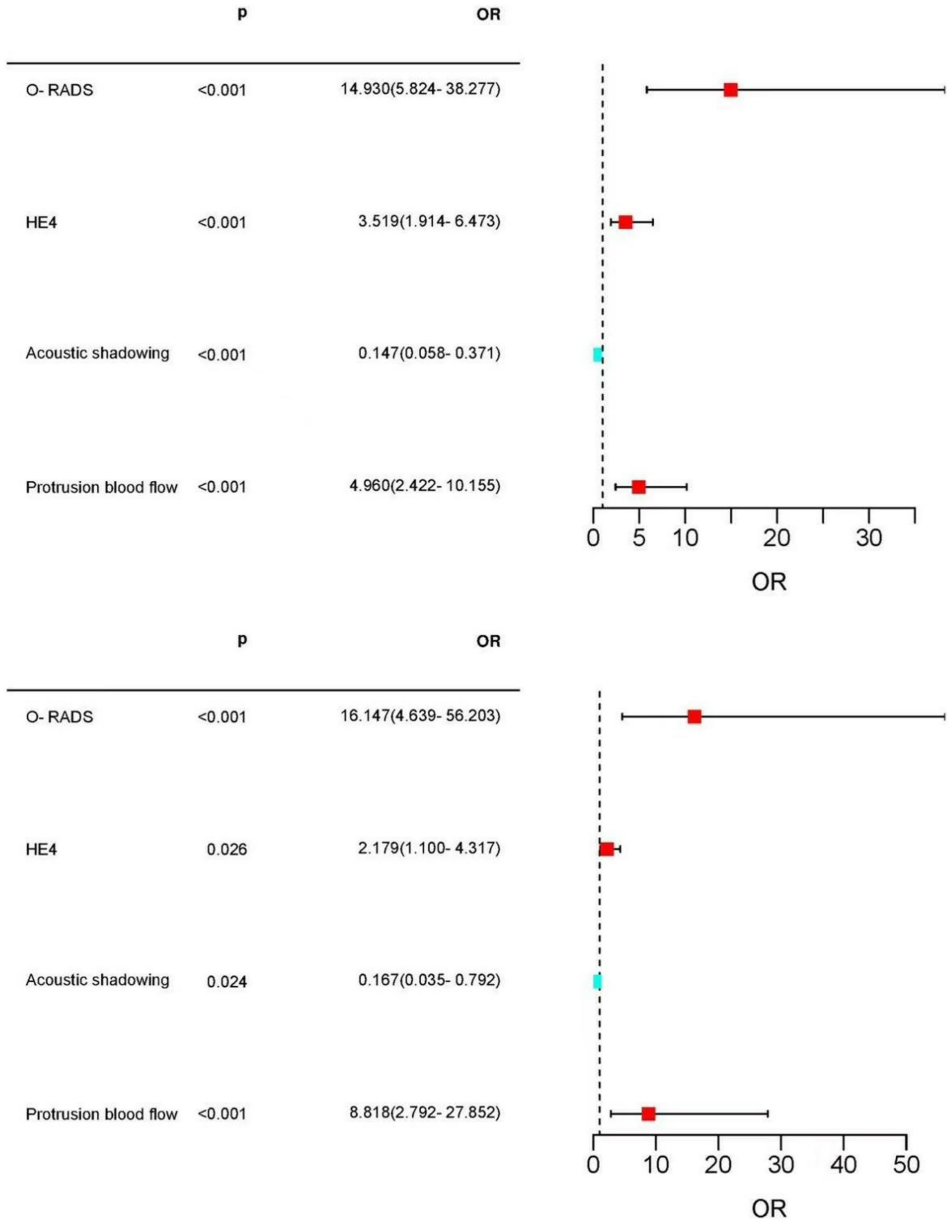
Forest plot of the selected feature. Forest plot used to visualize logistic regression analysis. O-RADS, HE4, protrusion blood flow score, and acoustic shadow were independent risk factors for adnexal malignancy. Acoustic shadows were a protective factor for malignant lesions in the adnexal region.

**Figure 4:**
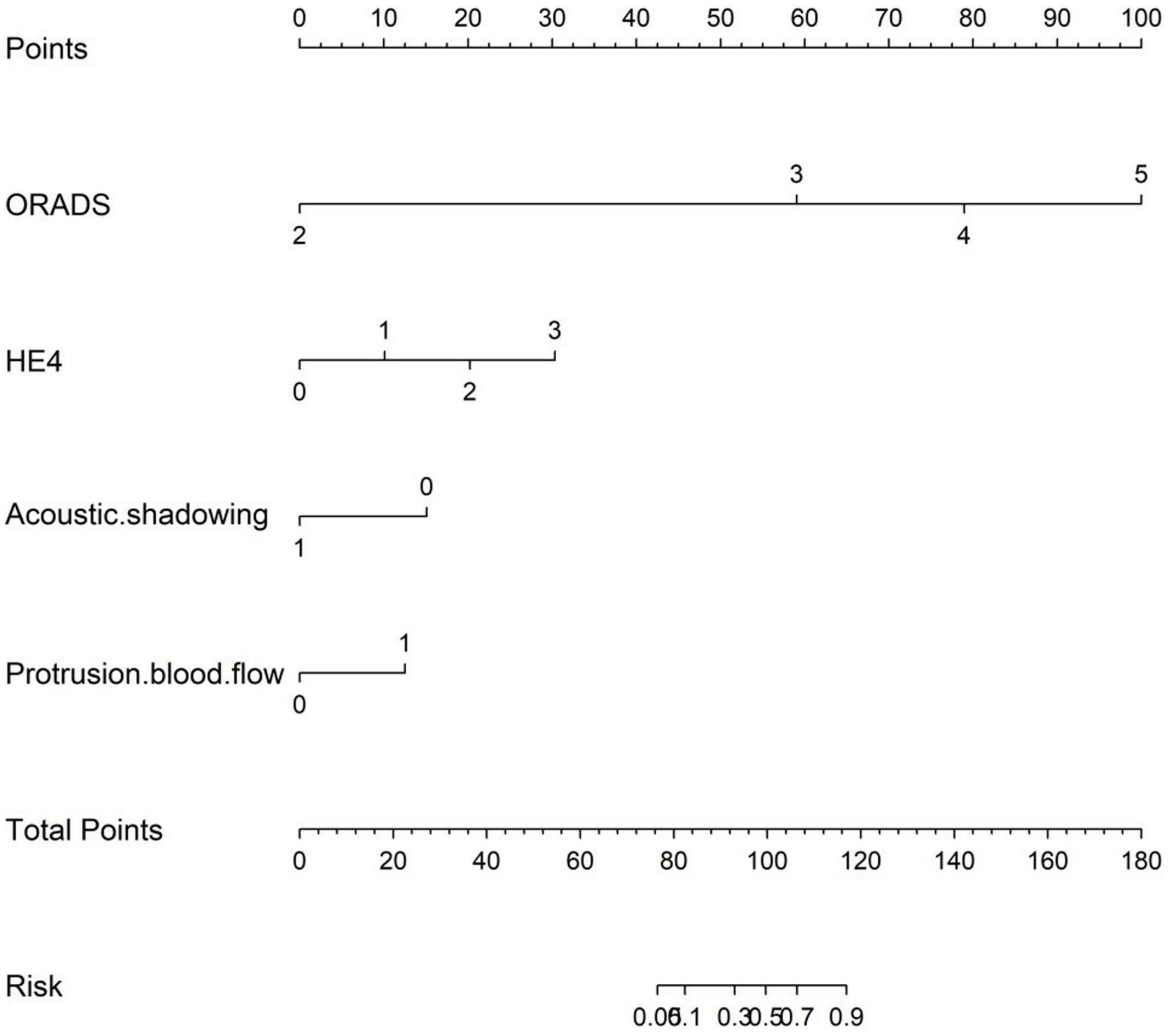
Nomogram for predicting the malignancy risk of complex ultrasound morphology masses. The probability of malignancy risk in adnexal masses is acquired by substituting the variables into the nomogram, drawing a vertical line, and finally giving the score of each variable and adding the scores of the four variables to obtain the total score.

Multifactorial logistic regression analysis showed that O-RADS (X1), HE4 levels (X2), acoustic shadow (X3), and blood flow scores of papillae (X4) were independent risk factors for adnexal malignant lesions. Fitting the regression equation to the risk factors for malignancy yielded Logit (P) = −11.528 + 2.703×1 + 1.258×2 + 1.601×3-1.918×4, and the ORs were 14.93, 3.519, 4.96, and 0.147, respectively (all *p* < 0.05, see Table 4). The binary logistic regression results are presented in the forest plot in Figures 2, 3. Establishing a nomogram on the basis of the multivariate logistic regression analysis (Figure 4).

**Table 4:** Multivariate logistic regression analysis to construct a nomogram model.

Malignant features (*p* < 0.05)	B*	SE*	OR*	P*	95%CI* Lower limit Upper limit	
O-RADS (X1)	2.70	0.48	14.93	0.00	5.82	38.28
HE4 (X2)	1.26	0.311	3.52	0.00	1.91	6.47
Protrusion blood flow (X3)	1.60	0.37	4.96	0.04	2.42	10.16
Acousticshadowing (X4)	−1.92	0.47	0.15	0.003	0.58	0.37

*B is the regression coefficient; SE is the standard error; OR is the ratio; P is the *p* value; 95% CI is the 95% confidence interval.

### 4.4 Model performance

**Figure 5:**
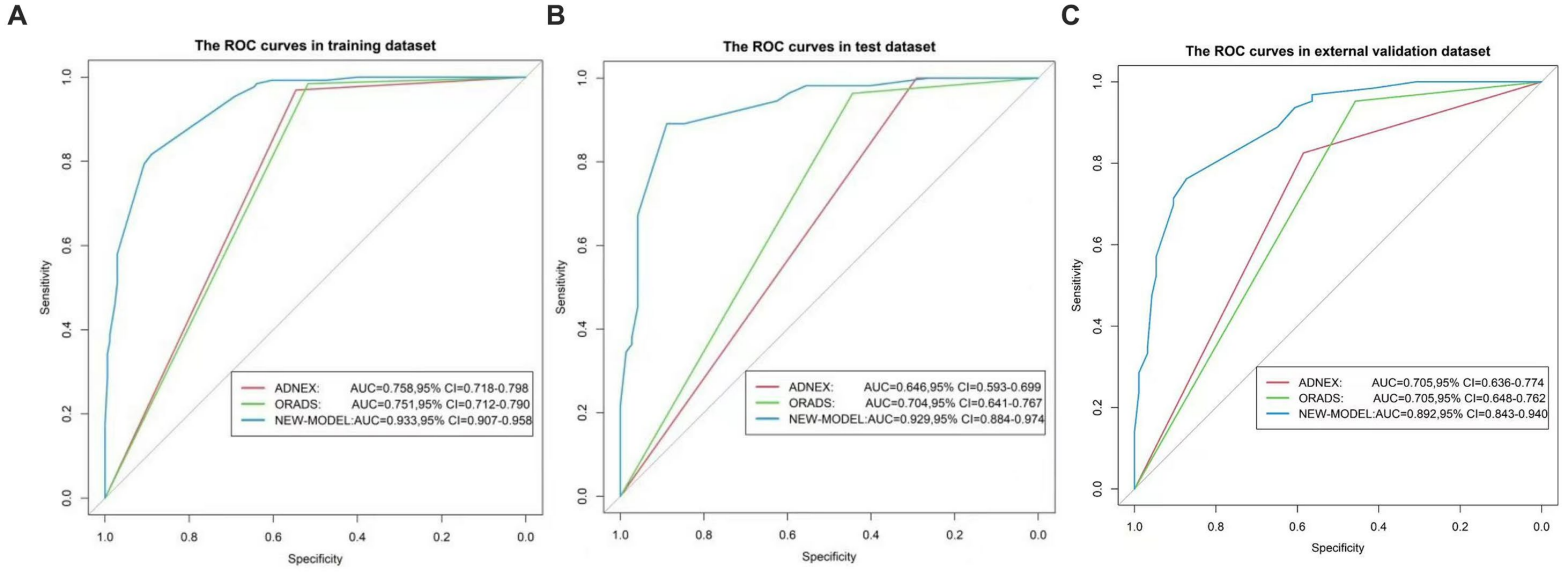
ROC curves of the nomogram. **(A)** Represents the training set. **(B)** Represents the validation set. **(C)** Represents the external validation set. The *x*-axis is the false-positive rate, while the *y*-axis is the true positive rate. The line shows the performance of the three models.

**Figure 6:**
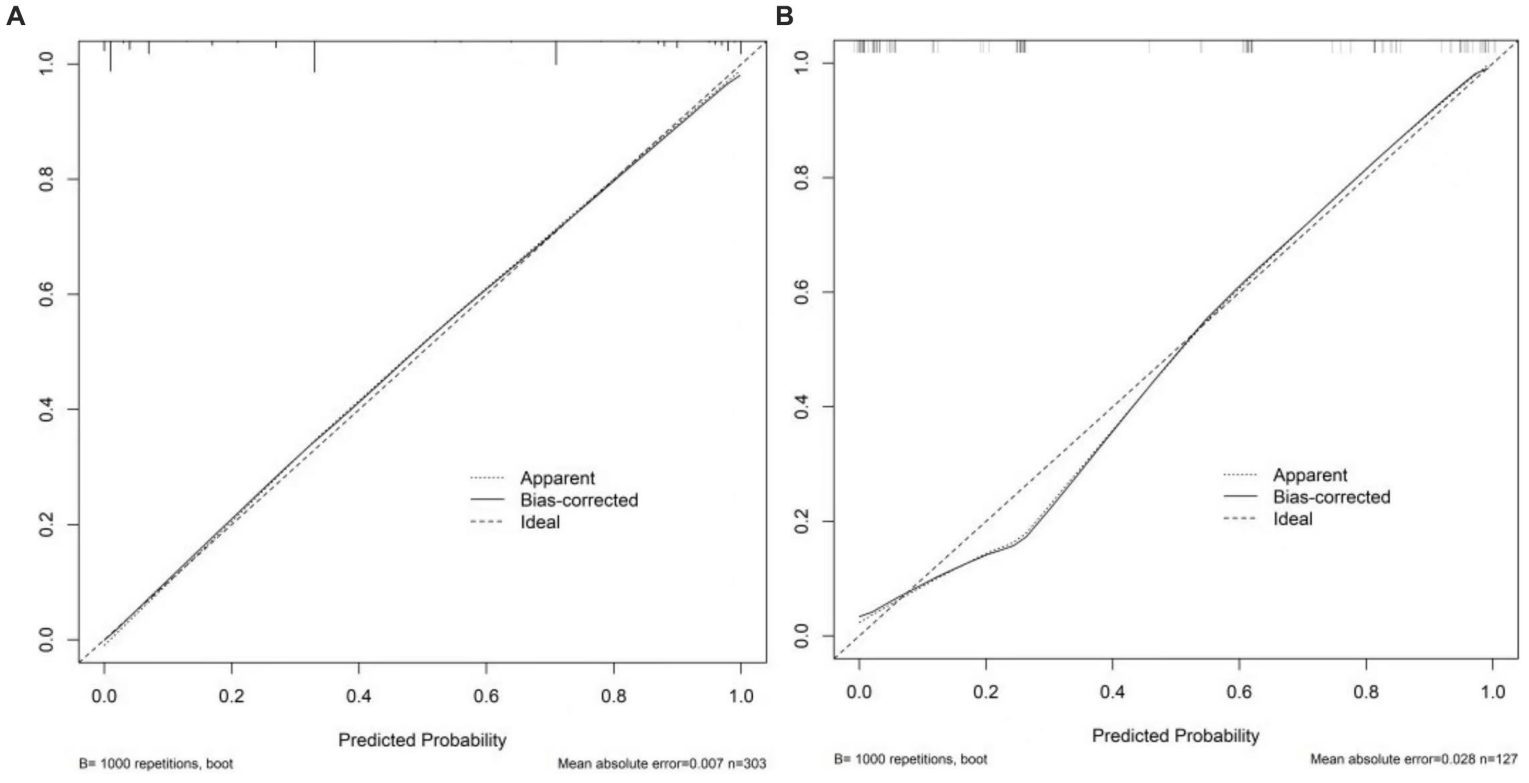
Calibration curves of the nomogram. **(A)** Stands for training set. **(B)** Stands for the validation set. The *x*-axis stands for the predicted probability of malignancy risk in adnexal masses. The *y*-axis stands for the actual diagnosed malignancy masses. The diagonal dotted line represents an ideal model for perfect prediction. The solid line stands for the performance of the nomogram. Closer fit to the diagonal dotted line represents a better prediction of the nomogram.

For the 430 patients in our study, the AUC value of the training set was up to 0.959 (95%CI:0.940–0.977), and the validation set was up to 0.929 (95%CI:0.884–0.974) (Figure 5). Both the training set and validation set showed excellent agreement in predicting complex lesions of calibration curves (Figure 6). It is suggested that the nomogram can forecast the incidence of complex lesions in the adnexal region accurately.

### 4.5 Clinical use

**Figure 7:**
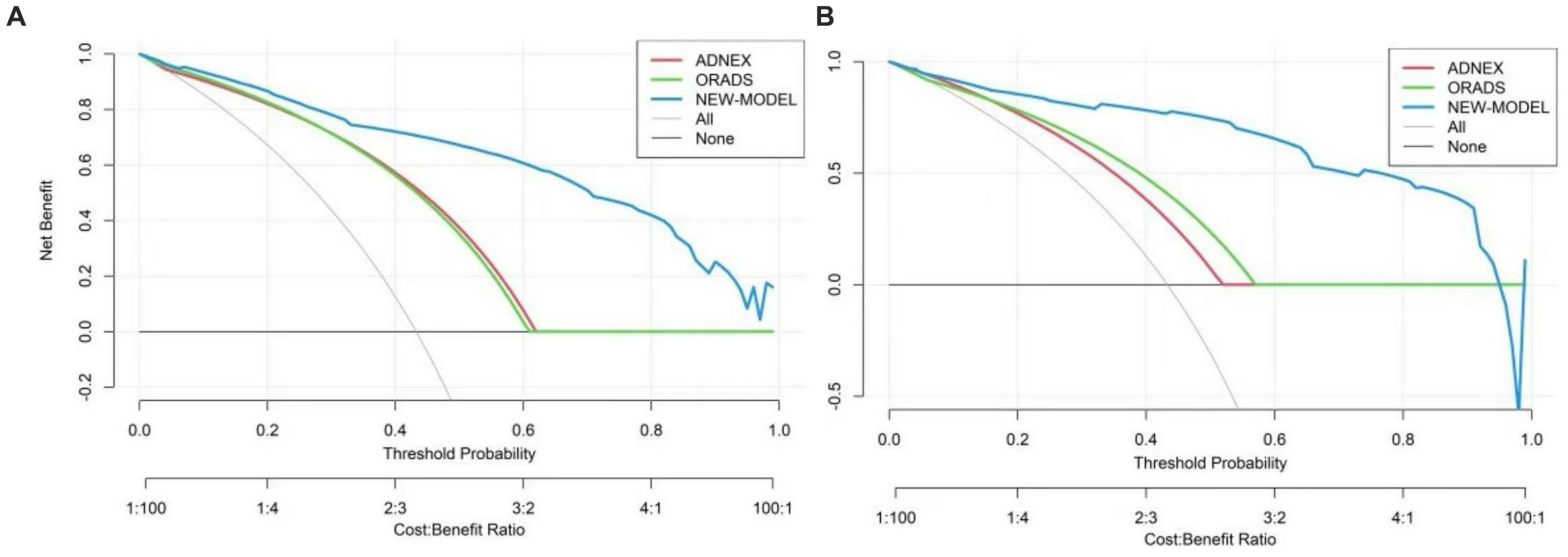
Decision curve analysis for the adnexal masses’ incidence risk nomogram. **(A)** Stands for the training set and **(B)** stands for the validation set. The *y*-axis represents the net benefit. The gray slash indicates the hypothesis that all patients were malignant, while the black solid line indicates the hypothesis that all patients do not get malignant tumors. The blue line stands for the risk nomogram.

**Figure 8:**
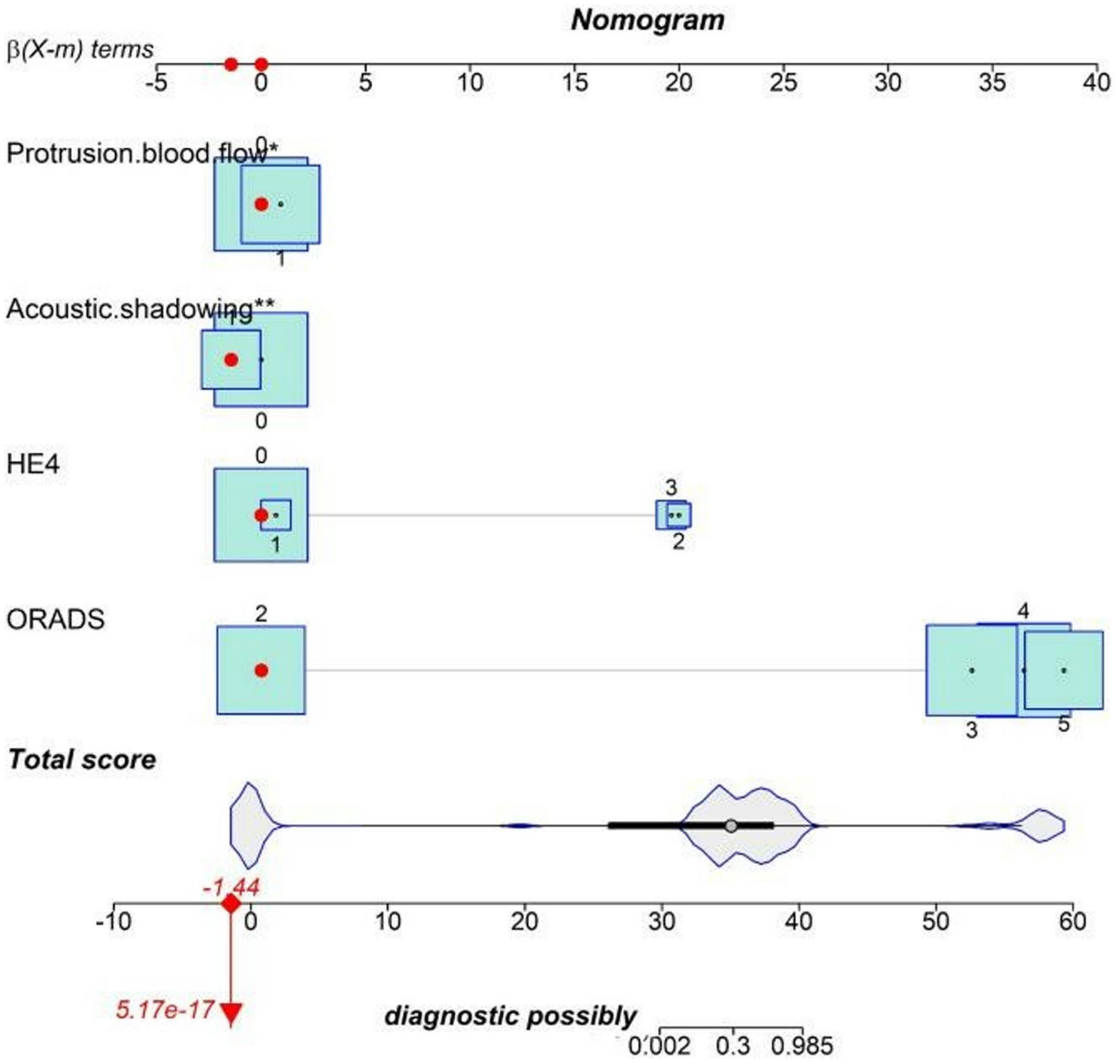
Dynamic nomogram. One subject was chosen from the population randomly selected by the characteristic indicators of the model. The indicators meant O-RADS = 2, HE4 level = 1-fold elevated, protrusion blood flow = no, and acoustic shadow = yes. The total score of the four variables shows that the predicted outcome is benign.

The nomogram was clinically beneficial in predicting the risk between a considerable range of threshold probabilities in the decision curve analysis (Figure 7). Thus, one subject was chosen at random from the population based on the characteristic indicators of the model. The indicators meant O-RADS = 2, HE4 level = 1-fold elevated, protrusion blood flow = no, and acoustic shadow = yes. We configured a dynamic nomogram to predict the frequency of complex lesions in the adnexal region (Figure 8).

### 4.6 Comparison of the diagnostic efficacy of the modified prediction model with O-RADS and ADNEX models

For the training set, the predictive model AUC = 0.933 (95% CI: 0.907, 0.958), the O-RADS classification system AUC = 0.751 (95% CI: 0.712, 0.79), and the ADNEX model AUC = 0.758 (95% CI: 0.718, 0.798). For the validation set, the prediction model AUC = 0.929 (95% CI: 0.884, 0.974), the O-RADS model AUC = 0.704 (95% CI: 0.641, 0.767), and the ADNEX model AUC = 0.646 (95% CI: 0.593, 0.699). The sensitivity, specificity, PPV, and NPV are summarized in Table 5.

**Table 5:** Sensitivity and specificity of three models.

Method	Sensitivity Specificity PPV NPV Accuracy AUC (95%CI)
IOTA-ADNEX model	
Training cohort	0.95 0.57 0.62 0.96 0.73 0.758 (0.718–0.798)
Validation cohort	1.0 0.30 0.52 1.0 0.60 0.646 (0.593–0.699)
External validation cohort	0.83 0.59 0.57 0.83 0.68 0.705 (0.636–0.774)
O-RADS model	
Training cohort	0.98 0.52 0.61 0.98 0.72 0.751 (0.712–0.79)
Validation cohort	0.96 0.44 0.57 0.94 0.67 0.704 (0.641–0.767)
External validation cohort	0.95 0.46 0.54 0.94 0.66 0.705 (0.648–0.762)
NEW-model	
Training cohort	0.83 0.89 0.89 0.86 0.86 0.933 (0.907–0.958)
Validation cohort	0.89 0.85 0.82 0.91 0.87 0.929 (0.884–0.974)
External validation cohort	0.81 0.83 0.89 0.80 0.84 0.892 (0.843–0.94)

### 4.7 External validation

Based on the external validation set of Harbin Medical University Cancer Hospital patients, further validation of the model’s predictive ability is needed. In the external validation set, AUC = 0.892 (95% CI: 0.843, 0.94) for the prediction model and AUC = 0.705 (95% CI: 0.648, 0.762) for the O-RADS model. The ADNEX model has an AUC of 0.705 (95% CI: 0.636, 0.774). Thus, our model has good clinical stability (Figure 5).

## 5 Discussion

The rising prevalence of adnexal masses showed that the task of preventing malignant tumor progression and delaying survival rates was daunting. Ultrasound was mostly classified as O-RADS III–IV, although ultrasound morphologic indexes were important references therein. The range of malignant intervals in the O-RADS classification system was too large due to the complex ultrasound manifestations of adnexal lesions (26), resulting in the generally low accuracy of the O-RADS used to evaluate adnexal zone lesions at present and a high rate of false positives (27). A diagnostic model based on ultrasound that combines multiple examination modalities has become a hot and difficult research topic at present. Thus, a diagnostic model was constructed to facilitate the early detection of intricate masses in the adnexal region based on the risk factors of ovarian cancer. In the present study, we found that O-RADS, HE4 levels, acoustic shadow, and protrusion blood flow were independent risk factors for adnexal malignant lesions. Many researchers have focused on the use of clinical models to predict adnexal zone lesions (12), and the incorporation of clinical history data and laboratory findings can reduce the subjectivity of stenographers in assessing adnexal zone masses. However, the assessment performance of clinical models in evaluating complex lesions in the adnexal region had rarely been recorded. Some scholars developed a nomogram to predict lesions in the adnexal region, and it identified race, surgery, chemotherapy, radiotherapy, age laterality, histology, stage, grade, and marital status as independent risk factors for ovarian cancer prognosis (28). Gong also developed a nomogram for predicting composite lesions in the ovary, which was validated in this study (13). The area under the ROC curve of Gong’s column line diagram was 89.8 and 91.2% in the training and validation sets, respectively, showing that the risk prediction model column line diagram we developed is more effective (Appendix 2). When compared to the traditional diagnostic model, our new nomogram can more accurately predict the classification of patient malignancy probability with complex ultrasound morphology masses, which is helpful for patient prognosis assessment and treatment strategy selection.

### 5.1 Risk factors for predicting complex lesions in the adnexal region

#### 5.1.1 O-RADS score

O-RADS US remained the most influential factor in the proposed predictive model, demonstrating the important role of standardized ultrasound terminology in O-RADS risk stratification when identifying the malignancy of ovarian lesions (29, 30). From the training cohort, this study found a sensitivity and specificity of 98.5 and 51.4%, respectively, for the O-RADS classification. This was comparable to those for O-RADS found by Hiett et al. (18) of 100 and 51.8% and Basha et al. (9) of 98.7 and 83.2%, respectively. The ADNEX model for diagnosing ovarian malignant lesions was 94.9 and 56.7%, respectively. With or without regard to the “inconclusive” lesions as malignant of the two models, the ADNEX demonstrated higher consistency under the ultimate pathology than the O-RADS model by using the 10% risk threshold. The ADNEX model showed a higher uniformity when compared to O-RADS (18) in the same 10% risk threshold.

#### 5.1.2 HE4 level

After adding CA125 as a laboratory index in the model, the diagnostic rate of ovarian malignant lesions improved. However, only 80% of epithelial ovarian cancers secreted CA125, and levels were easily affected by infection and pregnancy, resulting in a detection rate of less than 50% in the early stages of ovarian cancers. Meanwhile, CA125 levels appeared to be elevated in some benign ovarian diseases and other organ pathologies, for example, endometriosis cysts. The use of serum CA125 as a clinical indicator in the ADNEX model has been questioned because it reduces the sensitivity and specificity of CA125 in the diagnosis of ovarian cancer. Human epididymis gene product 4 (HE4) was a significant serologic indicator for the early diagnosis and differentiation of ovarian cancer (28). Several large-sample clinical studies have shown that HE4 has higher sensitivity and specificity than CA125 as a marker for ovarian cancer lesions (31), and greater sensitivity in the postoperative monitoring of ovarian cancer patients. Yang had previously shown that serum HE4 was an essential complementary indicator for CA125 (32). It had comparable sensitivity and high specificity.

#### 5.1.3 Protrusion blood flow

Kamel’s study showed that although papillae were included in O-RADS (33), they were easily missed because of their small size; many exogenic structures close to the inner wall of the cysts were often mistaken for papillae; and their morphology and color Doppler flow signals were more indicative of their significance (34). Cysts often present with irregularly thick walls (protrusion protuberances of <3 mm are defined as an irregular inner wall), and plasma papilloma might have single or multiple solid protrusions. Most of the pathologies of plasma papilloma were benign, and blood flow in the protrusion protuberances was a more precise indicator of the malignant potential of the protrusions, which was better represented by the abundance of the internal supplying blood vessels.

#### 5.1.4 Acoustic shadowing

The IOTA group was one of the first to utilize acoustic shadowing as a key feature in risk assessment tools for adnexal masses (35). The presence of acoustic shadowing greatly increased the likelihood that a mass would be benign, and Landolfo et al. (36, 37) indicated that acoustic shadowing was more likely to be present in benign lesions for complex unilocular cysts with a solid component and was common in dermatomes, plasma cysts, and fibromas. It was more likely that acoustic shadowing would be found in benign lesions and is often seen in dermatomes, plasma cysts, adenomas, and fibromas; therefore, acoustic shadows were included in this study model as a protective factor for malignant lesions.

#### 5.1.5 Other indicators

The risk of ovarian malignancy algorithm (ROMA) can be used to predict the occurrence of ovarian cancer-associated HE4 and CA125 levels according to menopausal status. The ROMA score was calculated based on the formula and expressed as a percentage rate corresponding to the predicted probability. In our study, the ROMA algorithm showed less specificity than that associated with HE4 levels (OR = 3.66 vs. OR = 4.26), but a better correlation than with CA125 levels (OR = 1.67 vs. OR = 3.66). The same study shows that ROMA was more sensitive than HE4, but with less specificity (38, 39). Contrarily, a study showed the limited value of detection based on CA125, HE4, and the ROMA algorithm as independent modalities for the prediction of early-stage adnexal malignant tumors and BOTs (40). Therefore, it was excluded from the model of our study. Previous literature suggests that HPV is not associated with the benign or malignant nature of ovarian tumors, but human papillomavirus (HPV) was a high-risk etiological factor for ovarian cancer in Northeast China, whose prevalence varied by geographic region (41, 42). Given the background, our study took HPV infection into account. However, this indicator was not statistically significant in this study. Ascites is a hallmark of ovarian cancer, and this remarkable fluid presents in advanced ovarian cancer and peritoneal implantation metastases (43). Few of the cases included in our study involved advanced ovarian cancer, and ascites could also be found in other diseases, such as patients with ovarian lesions combined with advanced ascites in cirrhosis, resulting in its specificity being obscured. Therefore, ascites was of little significance in this model.

Three models (the risk prediction, ADNEX, and O-RADS US models) were validated in this study to assess complex lesions in the adnexal area with separate algorithms. The results of the training and validation sets show both the ADNEX and the O-RADS models perform well, and the prediction model in this study improved the detection rate of compound atypical lesions without compromising the sensitivity. Compared with other ultrasound diagnostic models, the comprehensive prediction model in this study had higher diagnostic efficacy and clinical application value than other models and could therefore provide competitive clinical risk assessment value. The stability of our model was further demonstrated in the external validation.

Our study had a lot of strengths. First, for the prediction model, our study obtained comprehensive patient information in our hospital system. The information from the ADNEX model and O-RADS US were all used with the same cohort of patients, making the assessment results more comparable (10, 44). We comprehensively analyzed the factors affecting the malignancy of complex lesions in the adnexal region and established a nomogram to train and validate the sensitivity, specificity, and clinical value of this predictive model. Second, the two validation cohorts from different centers allowed us to validate our results (45). The AUCs of the predictive model were all higher than those of the traditional metrics, which represents the stability and broad consistency of the model across cohorts. Third, the metrics included in this study are novel, and there are no relevant models that use metrics such as the ones above. However, our study still had some limitations. First, selective bias and inherent errors were inevitable in a retrospective study. Second, although there was a high degree of agreement between physicians on O-RADS scores, subjective constraints made human bias unavoidable. Third, a lack of surgical inpatients prevented knowledge of low-risk or screened patients from different geographic regions, which may lead to an imbalance of malignant risk in the statistics of the O-RADS classification system. Finally, despite the predictive model’s ability to effectively discriminate between benign and malignant adnexal zone lesions and to suggest more refined management options, larger prospective trials are needed in order to validate this observation.

## 6 Conclusion

In conclusion, the risk prediction model proposed in this study had high diagnostic accuracy in differentiating complex adnexal lesions, and the model is still based on the O-RADS classification system, which makes our model more convincing. Then, additional management of complex atypical lesions was more conducive to hospital-stratified management and screening triage and improved patient prognosis. Therefore, our model could potentially serve as a non-invasive approach to assessing complex adnexal lesions, assisting in the development of personalized treatment strategies, guiding ovarian mass management, and probably avoiding unnecessary puncture biopsy. Further studies, such as deep learning and prospective studies, should be carried out, which could determine the clinical feasibility of our predictive model.

## Data availability statement

The raw data supporting the conclusions of this article will be made available by the authors, without undue reservation.

## Ethics statement

The studies involving humans were approved by Clinical Trial Ethics Committee of the Fourth Affiliated Hospital of Harbin Medical University (No.: 2023-SCILLSC-14). The studies were conducted in accordance with the local legislation and institutional requirements. The participants provided their written informed consent to participate in this study. Written informed consent was obtained from the individual(s) for the publication of any potentially identifiable images or data included in this article.

## Author contributions

YW: Conceptualization, Data curation, Formal analysis, Investigation, Methodology, Resources, Supervision, Writing – original draft, Writing – review & editing. KM: Conceptualization, Methodology, Software, Visualization, Writing – review & editing. TW: Investigation, Supervision, Validation, Writing – review & editing. CX: Formal analysis, Resources, Writing – review & editing. JY: Conceptualization, Supervision, Writing – original draft. XD: Data curation, Funding acquisition, Resources, Writing – original draft.

## Footnotes

^1^ https://www.iotagroup.org/iota-models-software/adnex-risk-model
