# Supplementary material for: Prediction model of adnexal masses with complex ultrasound morphology
Source: Front Med (Lausanne). 2023 Dec 7;10:1284495. doi: 10.3389/fmed.2023.1284495 (PMC10740199; doi:10.3389/fmed.2023.1284495)
Supplement: Supplementary file 1 [file Image_1.pdf]

Supplementary materials

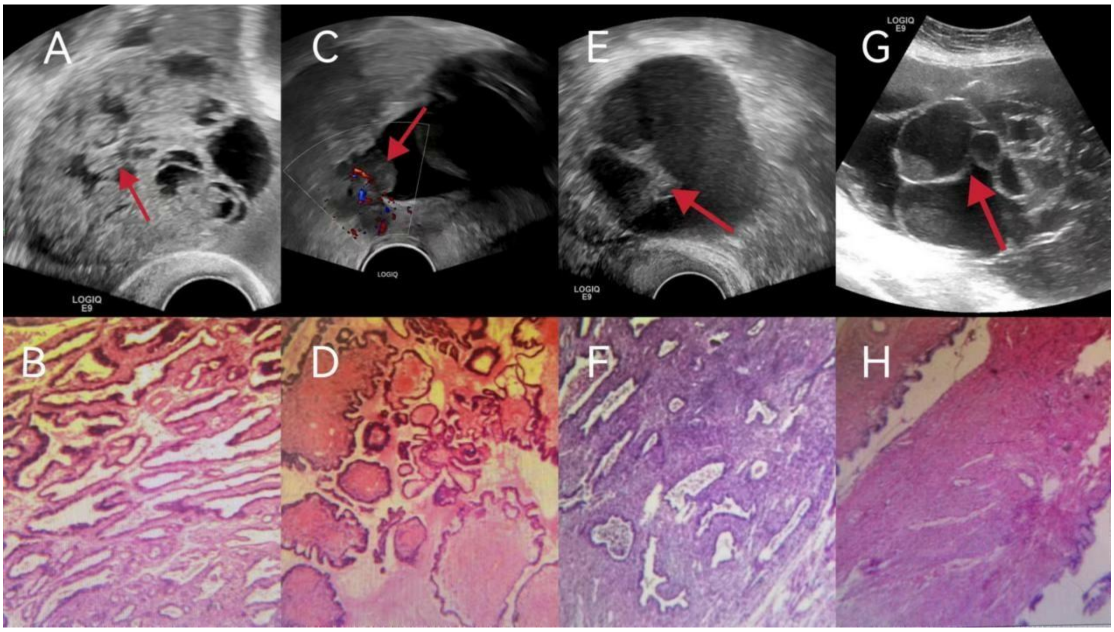

Appendix 1: The ultrasound image shows a nearly circular cystic solid mass, predominantly solid with protrusions and separation (A, arrows); the pathological image proves to be a malignant metastatic Kubo's tumor(B)； D is a junctional high-grade plasmacytoma which shows that a hypoechoic lesion with a solid component visible on one side and CDFI which shows a large amount of blood flow signal within the tumor (C, arrows); F is a luteal hematomas which shows that a cystic predominantly mixed echogenic light mass with flocculent echogenicity(E, arrow).H is a typical sonogram of mucinous cystadenoma shows that a cystic light mass with poor transmission and multiple compartments separated within(G, arrow).

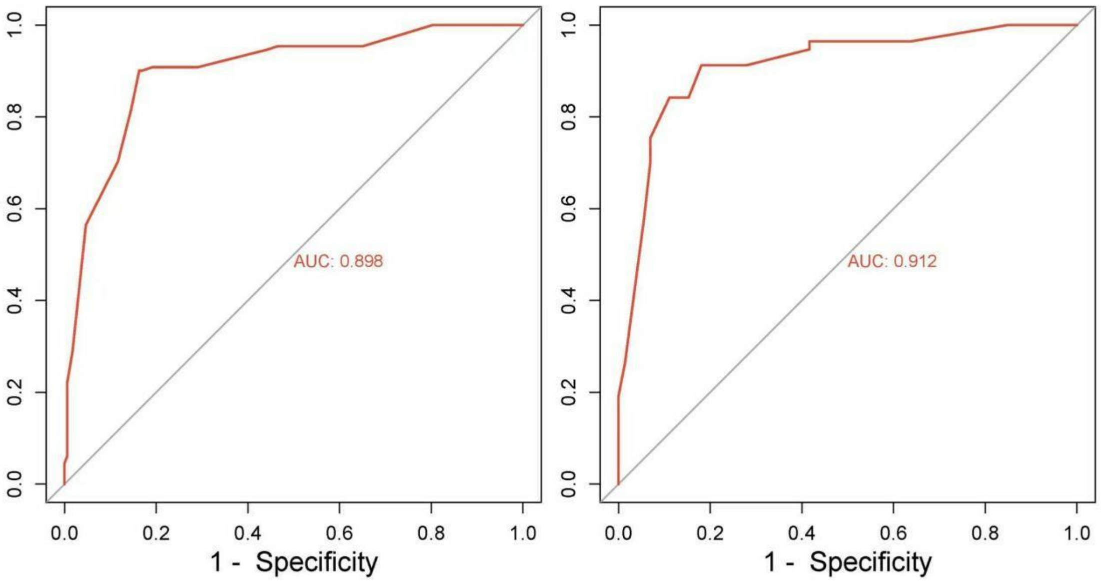

Appendix 2: Gong's Nomogram ROC Curve.
The left side is the training set in our study; The right side is the validation set.
